# Draft genome sequence of *Lactiplantibacillus plantarum* subsp. *plantarum* strain HF43, a human gut-associated potential probiotic

**DOI:** 10.1128/MRA.00945-22

**Published:** 2023-07-19

**Authors:** Maryam Idrees, Naima Atiq, Rabaab Zahra, Muhammad Imran, Shakira Ghazanfar

**Affiliations:** 1 Department of Microbiology, Quaid-i-Azam University, Islamabad, Pakistan; 2 National Institute for Genomics and Advanced Biotechnology (NIGAB), National Agricultural Research Centre, Islamabad, Pakistan; University of Maryland School of Medicine, Baltimore, Maryland, USA

**Keywords:** *Lactiplantibacillus plantarum* subsp. *plantarum*, human gut associated, target-based probiotics, draft genome, probiotics

## Abstract

*Lactiplantibacillus plantarum* adapts to a wide range of ecological niches, including the human gut. Numerous health-promoting benefits have been associated with *L*. *plantarum* strains. Motivated for the development of human-origin target-based probiotics with known genetic markers, we report the draft genome sequence of human gut-associated *Lactiplantibacillus plantarum* subsp. *plantarum* HF43.

## ANNOUNCEMENT

*Lactiplantibacillus plantarum* exhibits the capability of inhabiting an extensive range of ecological niches, including the human gastrointestinal tract (1 - 3). It is a constituent of phylum Firmicutes, one of the two major phyla that dominate the gut microbiome (4). *L. plantarum* is identified to restore the intestinal mucosal barrier (5), regulate dysbacteriosis, reduce inflammation, and alleviate inflammatory bowel disease (6, 7). Moreover, *L. plantarum* has antimicrobial, antioxidant, and anticancer potential (8). The advances in multi-omics have led to probiogenomics, an approach to identify genetic markers that facilitate the adaptation of a probiotic to human gut (9, 10). Omics data expedite the formulation of target-based probiotics by identifying molecular effectors that positively regulate host pathways to promote health-associated phenotypes (11 - 13). In recent years, the use of human-origin probiotics has been favored over non-human lineage probiotics as host-adapted strains carry niche-specific genetic modifications (11, 14 - 17); ergo, human-origin strains exhibit superior performance in contrast to plant- and dairy-derived probiotics (18). Here, we present findings for *L. plantarum* subsp. *plantarum* HF43, a potential probiotic isolated from the human gut.

The fecal sample was collected and processed at the Department of Microbiology, Quaid-i-Azam University, Islamabad, Pakistan, in October 2019, accredited by Bio-Ethical Committee (BEC) of Quaid-i-Azam University under approval number BEC-FBS-QAU2021-333. Fecal sample was serially diluted and cultured on De Man, Rogosa, & Sharpe (MRS) agar medium and incubated at 37°C for 24–48 hours. A single bacterial colony was selected and cultured in MRS broth and preserved in glycerol at −80°C. A volume of 30 µL from this master stock was independently cultured on MRS agar for further use. The genus and species were determined by 16S rDNA sequencing by protocol detailed elsewhere (19). DNA was isolated by phenol-chloroform method (20). Library preparation and sequencing were performed by Azenta Life Sciences. DNA Library was prepared with Covaris 220, and sequencing was performed with Illumina HiSeq 4000 platform (2 × 150-bp reads). Raw reads were trimmed with fastp v0.23.1. This workflow produced a total of 3,457,873 trimmed reads. Raw reads were assembled with Unicycler v0.4.8, and coverage was estimated with BBMap v38.47. The resulting contigs were subjected to ORF prediction and annotation using RAST v2.0 (21) and the NCBI Prokaryotic Genome Annotation Pipeline (PGAP) v6.5 (22). Unless specified otherwise, default parameters were used for all software tools.

The *L. plantarum* subsp. *plantarum* HF43 genome is comprised 12 contigs corresponding to 3,121,984 bp (∼10× coverage), a GC content of 44.8% and *N*_50_ value of 1,852,232 bp. PGAP predicted 2,934 genes, representing 2,747 protein coding sequences, 5 5S rRNA sequences, 4 16S rRNA sequences, 4 23S rRNA sequences, 64 tRNA sequences, and 4 ncRNA sequences. Our findings are concordant with already published human gut-associated *L. plantarum* genomes (3, 23).

## Data Availability

This genome project is indexed at GenBank under BioProject accession number PRJNA773595 (24). The genome sequence for *Lactiplantibacillus plantarum* subsp. *plantarum* HF43 is deposited to GenBank under accession number NZ_JALGYB000000000, and the reported genome is the first version, NZ_JALGYB000000000.1 (25). Trimmed Illumina raw sequence reads have been deposited under accession number SRR24481500 (26).
